# Stoichiometric optimization of Gata4, Hand2, Mef2c, and Tbx5 expression for contractile cardiomyocyte reprogramming

**DOI:** 10.1038/s41598-019-51536-8

**Published:** 2019-10-18

**Authors:** Zhentao Zhang, Wenhui Zhang, Young-Jae Nam

**Affiliations:** 1 Department of Medicine, Division of Cardiovascular Medicine, Vanderbilt University Medical Center, Nashville, TN USA; 2 Department of Cell and Developmental Biology, Vanderbilt University, Nashville, TN USA; 3 Vanderbilt Center for Stem Cell Biology, Vanderbilt University, Nashville, TN USA

**Keywords:** Reprogramming, Cardiac regeneration

## Abstract

Reprogramming of fibroblasts to induced cardiomyocyte-like cells (iCMs) offers potential strategies for new cardiomyocyte generation. However, a major challenge of this approach remains its low efficiency for contractile iCMs. Here, we showed that controlled stoichiometric expression of Gata4 (G), Hand2 (H), Mef2c (M), and Tbx5 (T) significantly enhanced contractile cardiomyocyte reprogramming over previously defined stoichiometric expression of GMT or uncontrolled expression of GHMT. We generated quad-cistronic vectors expressing distinct relative protein levels of GHMT within the context of a previously defined splicing order of M-G-T with high Mef2c level. Transduction of the quad-cistronic vector with a splicing order of M-G-T-H (referred to as M-G-T-H) inducing relatively low Hand2 and high Mef2c protein levels not only increased sarcomeric protein induction, but also markedly promoted the development of contractile structures and functions in fibroblasts. The expressed Gata4 and Tbx5 protein levels by M-G-T-H transduction were relatively higher than those by transductions of other quad-cistronic vectors, but lower than those by previously defined M-G-T tri-cistronic vector transduction. Taken together, our results demonstrate the stoichiometric requirement of GHMT expression for structural and functional progresses of cardiomyocyte reprogramming and provide a new basic tool-set for future studies.

## Introduction

The adult human heart cannot regenerate heart muscle after injury, due to its very limited regenerative capacity. Thus, regenerating new heart muscle cells has been a highest research priority in the field. In this regard, directly reprogramming fibroblasts to induced cardiomyocyte-like cells (iCMs) by forced expression of cardiogenic factors (referred to as cardiomyocyte reprogramming) may provide an entirely new approach to generate new cardiomyocytes for various clinical applications. Since Srivastava’s group first demonstrated the proof of this concept^1^, numerous progresses have been made to improve cardiomyocyte reprogramming efficiency and quality^2^. In addition, the recent study showed that directly reprogrammed iCMs are more mature than pluripotent stem cell derived cardiomyocytes^3^. However, the total number of contractile iCMs generated by this approach is relatively small. Following transduction of cardiogenic reprogramming factors, a major population of fibroblasts fail to develop contractile structures and functions, although they induce cardiac proteins, which are normally absent in fibroblasts^4^. These findings suggest that there exists a barrier between inducing cardiac proteins and developing contractile structures composed of induced cardiac proteins during fibroblast to cardiomyocyte reprogramming. Only a fraction of the fibroblasts expressing cardiac proteins may stochastically overcome this barrier and thus adopt a contractile phenotype.

We recently demonstrated that ensuring expression of four cardiogenic transcription factors (i.e. Gata4 (G), Hand2 (H), Mef2c (M), and Tbx5 (T); referred to as GHMT) in individual fibroblasts is an initial necessary step for the efficient reprogramming process toward contractile iCMs^5^. While the combination of three cardiogenic transcription factors (i.e. Gata4, Mef2c, and Tbx5; referred to as GMT) was sufficient to induce sarcomere proteins in ~70–80% of the fibroblasts expressing GMT similar to GHMT, adding Hand2 to GMT markedly increased the number of fibroblasts displaying contractile structures and functions over GMT alone. However, only a relatively small fraction of fibroblasts expressed all four cardiogenic transcription factors following transduction of pooled viruses expressing individual factors^5^. Therefore, we sought to develop a transduction method which can ensure expression of all four cardiogenic transcription factors in individual fibroblasts. Among several strategies for simultaneously expressing multiple genes using a single plasmid, a self-processing 2A peptide-based approach appears most suitable for expressing as many as four factors due to the short length of 2A peptides (18–22 amino acids)^6,7^. Importantly, the previous study using this 2A peptide-based expression system demonstrated that relatively higher expression of Mef2c in conjunction with lower expression of Gata4 and Tbx5 significantly enhanced cardiomyocyte reprogramming efficiency and quality^8^. In that study, relative protein expression levels of GMT were manipulated by a relative position of each factor in tri-cistronic vectors in which each cardiogenic gene is linked by a 2 A peptide. The gene positioned relatively upstream expressed higher protein level than the one relatively positioned downstream in polycistronic constructs^7,8^. Based on the importance of secure expression of all four reprogramming factors (i.e. GHMT)^5^ as well as stoichiometric expression of GMT for cardiomyocyte reprogramming^8^, we hypothesized that optimizing stoichiometric expression of GHMT using a quad-cistronic expression system will further enhance cardiomyocyte reprogramming.

To test our hypothesis, we have generated four different quad-cistronic constructs containing GHMT in which *Hand2* is placed at four different positions while maintaining relative splicing order of M-G-T leading to high Mef2c level based on the previous studies (i.e. M-G-T-H, M-G-H-T, M-H-G-T, and H-M-G-T)^7,8^. We found that Hand2 protein expression level induced by each quad-cistronic vector is significantly altered, depending on its relative position to other factors in each construct. We demonstrated that M-G-T-H transduction inducing relatively lower protein level of Hand2 in the context of high Mef2c protein level in fibroblasts substantially increased the number of fibroblasts adopting distinctive contractile phenotypes of a cardiomyocyte (i.e. sarcomeric organization, calcium flux, and spontaneous contraction) beyond sarcomeric protein expression. These findings provide new mechanistic insights into structural and functional maturation of iCMs and an important genetic tool which could be universally used for future cardiomyocyte reprogramming studies.

## Results

### Protein expression levels of Gata4, Hand2, Mef2c, and Tbx5 by transduction of four different quad-cistronic constructs

**Figure 1:**
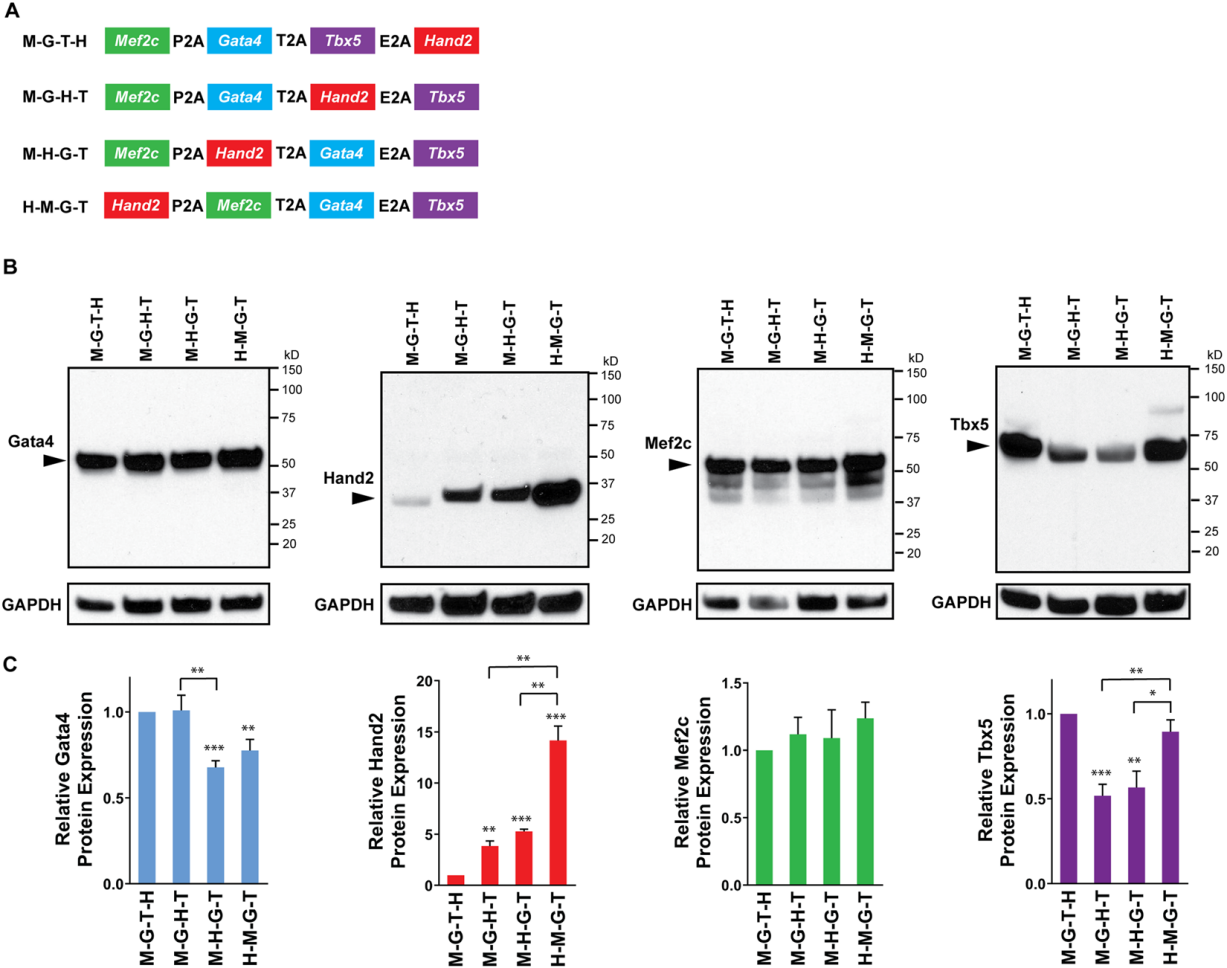
Protein expression of cardiogenic transcription factors harbored in quad-cistronic constructs. (**A**) Schematic illustration of four distinct quad-cistronic constructs encoding GHMT with different splicing orders. (**B**) Western blot analysis for GHMT protein expression. Three days after transducing indicated quad-cistronic vectors encoding GHMT into MEFs, cell lysates were collected. An arrow head indicates a protein band of each cardiogenic transcription factor. (**C**) Quantification of relative GHMT protein expression levels. Six independent experiments are presented as mean ± s.d. *P < 0.05; **P < 0.01; ***P < 0.0001 versus M-G-T-H unless specifically indicated.

To manipulate protein expression level of Hand2 within the context of previously optimized stoichiometric expression of GMT (splicing order of M-G-T)^8^, we generated four different quad-cistronic constructs containing GHMT in which *Hand2* is placed in four different positions while relative positions of GMT remains the same (M-G-T) (i.e. M-G-T-H, M-G-H-T, M-H-G-T, and H-M-G-T) as illustrated in Fig. 1A. We used the combination of three different 2A peptides (P2A-T2A-E2A) to link individual four factors in quad-cistronic vectors, because a quad-cistronic vector containing three different 2A peptides in the order of either P2A-T2A-E2A or T2A-P2A-E2A was shown to be effective for protein expression of all four encoded genes^7^. We wished to induce different levels of Hand2 protein while maintaining similar relative protein levels of GMT as previously defined for optimal cardiomyocyte reprogramming using tri-cistronic vectors^8^. We transduced these vectors into mouse embryonic fibroblasts (MEFs) and analyzed protein expression levels of individual factors using western blotting (Fig. 1B,C). Gata4 protein expression by M-G-T-H and M-G-H-T constructs harboring *Gata4* at the second position from 5′ end was higher than the one by M-H-G-T and H-M-G-T constructs, where *Gata4* was located at the third position from 5′ end. Hand2 protein levels significantly changed depending on the position of *Hand2* in the quad-cistronic constructs. H-M-G-T construct harboring *Hand2* at 5′ end showed the highest Hand2 expression, while M-G-T-H constructs encoding *Hand2* at 3′ end revealed the lowest expression. There was no significant difference in Mef2c protein levels among all four constructs. Although H-M-G-T construct harbors *Tbx5* at 3′ end, it demonstrated significantly higher Tbx5 expression level than other constructs harboring *Tbx5* at 3’ end (i.e. M-G-H-T and M-H-G-T) (Fig. 1B,C). We also compared Gata4, Mef2c, and Tbx5 protein levels expressed by M-G-T-H transduction with those by M-G-T transduction (Fig. S1). While Mef2c protein expression by M-G-T-H transduction is not significantly different from the one by M-G-T transduction, both Gata4 and Tbx5 protein expression levels were modestly lower in M-G-T-H transduced cells than in M-G-T transduced cells.

### Enhancing sarcomeric protein induction by stoichiometric alteration of GHMT expression

**Figure 2:**
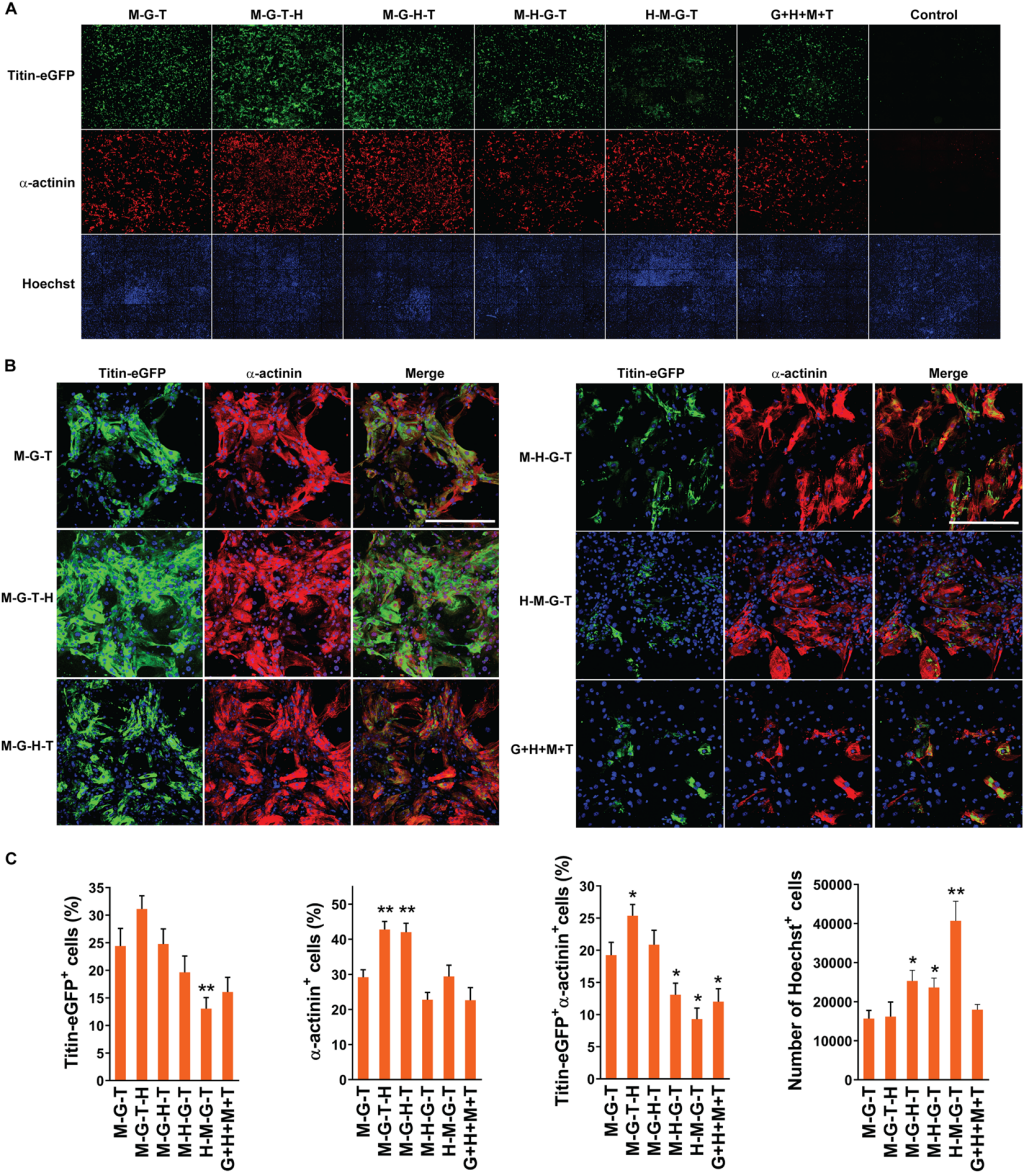
Quantification of sarcomeric protein induction using high content imaging analyses. (**A**) Representative composite immunofluorescent images used for high content imaging analyses to quantify Titin-eGFP and α-actinin expression following indicated transductions. The indicated constructs were transduced into MEFs isolated from *Titin-eGFP* reporter knock-in mice. Immunofluorescence staining for eGFP and α-actinin followed by high content imaging analyses was performed at day 15 post-transduction. Nuclei are stained with Hoechst. Each panel shows a composition of 25 images taken by the high content imaging system using a 10X objective. (**B**) Induction of sarcomeric proteins (i.e. Titin and α-actinin) following transduction of indicated constructs. A single representative immunofluorescent image used for high content imaging analyses following indicated transductions was shown. Scale bar, 400 µM. (**C**) Summary of high content imaging analyses for the percentage of Titin-eGFP^+^ and/or α-actinin^+^ cells or the number of Hoechst^+^ cells. Twelve independent experiments are presented as mean ± s.d. *P < 0.05; **P < 0.01 versus M-G-T.

As an initial reprogramming process of fibroblasts toward a cardiac fate, we analyzed induction of sarcomeric proteins using a high content imaging analysis (ImageXpress Micro High-Content Imaging Systems, Molecular Devices) as we previously described^5^. We transduced MEFs isolated from *Titin-eGFP* reporter knock-in mice^9^ with four quad-cistronic constructs (i.e. M-T-G-H, M-T-H-G, M-H-G-T, and H-M-G-T), the M-G-T tri-cistronic construct, or individual constructs encoding each of GHMT together. To enhance cardiomyocyte reprogramming, we cultured transduced MEFs in the induction media containing Tgf-β inhibitors (i.e. A-83-01 and SB431542) throughout this study^10,11^. At day 15 post-transduction, we immunostained cells for eGFP and α-actinin and quantified the percentage of cells expressing Titin-eGFP and/or α-actinin using a high content imaging system (Fig. 2A). We found that M-G-T-H transduction demonstrated the highest induction efficiency of Titin-eGFP^+^, α-actinin^+^ and Titin-eGFP^+^α-actinin^+^ cells among all tested transduction recipes (Fig. 2B,C). However, there was no statistically significant difference in Titin-eGFP induction efficiency between M-G-T-H and M-G-T or M-G-H-T transductions. Although M-G-H-T transduction significantly increased induction efficiency of α-actinin^+^ cells, it did not enhance that of Titin-eGFP^+^ or Titin-eGFP^+^α-actinin^+^ cells over M-G-T transduction. Among all quad-cistronic constructs tested, M-G-T-H construct demonstrated the lowest expression of Hand2 with relatively higher expression of Gata4 and Tbx5 (Fig. 1C). Compared to M-G-T-H transduction, M-G-H-T transduction showed higher level of Hand2 and lower level of Tbx5. While relatively lower Gata4 and Tbx5 protein levels in the context of tri-cistronic GMT expression were shown to be beneficial for cardiomyocyte reprogramming along with higher Mef2c level^8^, relatively higher Gata4 and Tbx5 protein levels with the similarly high level of Mef2c in the context of quad-cistronic GHMT protein expression increased sarcomere protein induction in fibroblasts (Figs 1C and 2C). By directly comparing the protein levels between M-G-T-H and M-G-T transductions, we showed that M-G-T-H induced Gata4 and Tbx5 protein levels, which were relatively higher than the levels expressed by other quad-cistronic constructs, were actually lower than those induced by tri-cistronic M-G-T transduction displaying relatively lower Gata4 and Tbx5 expression compared to transductions of other tri-cistronic vectors^8^ (Fig. S1). These results suggest that a narrow range of Gata4 and Tbx5 protein expression levels may be required for efficient fibroblast to cardiomyocyte reprogramming. Taken together, we demonstrated that induction of sarcomere proteins in fibroblasts can be promoted by controlling relative protein levels of four reprogramming factors (i.e. GHMT) using a quad-cistronic vector system.

We further tested whether M-G-T-H transduction is superior to combinatorial transduction of tri-cistronic M-G-T vector plus Hand2 mono-cistronic vector (referred to as M-G-T + H) which showed enhanced cardiomyocyte reprogramming over transduction of M-G-T alone in the recent study^12^. Following transduction of indicated vectors, we analyzed cardiac troponin T (cTnT) expression using flow cytometry (Fig. S2). Although the difference was modest, M-G-T-H transduction induced significantly higher percentage of cTnT expressing cells than M-G-T + H. Consistent with the previous study^12^, M-G-T + H was superior to M-G-T alone for sarcomere protein induction in fibroblasts. It is important to point out two possible reasons for less efficient reprogramming by M-G-T + H than M-G-T-H. First, relative Hand2 protein level in individual cells transduced by M-G-T + H is likely stochastic rather than controlled, given that retroviruses randomly integrate into the infected cell’s genome. Only a small fraction of transduced cells may present an optimal relative expression ratio of Hand2 protein. Second, a significantly smaller fraction of whole cell population expresses all four factors following transduction of two vectors (i.e. M-G-T plus Hand2), compared to M-G-T-H single vector transduction as we recently demonstrated^5^. In other words, a significant fraction of cells may express M-G-T or Hand2 alone following M-G-T + H transduction. Taken together, these results re-emphasize the importance of optimal stoichiometric expression of Hand2 as well as ensuring expression of all desired factors for efficient fibroblast to iCM conversion.

Interestingly, number of nuclear staining positive cells are markedly higher with H-M-G-T transduction, which showed lower reprogramming efficiency compared with other groups (Fig. 2C). Although it is unclear whether less-inhibited cell proliferation is a cause or consequence of low reprogramming efficiency by H-M-G-T transduction, these results are consistent with the recent study demonstrating that cardiomyocyte reprogramming is inversely correlated with cell proliferation^12^. In contrast, M-G-T or M-G-T-H transduction showed the lowest number of Hoechst positive cells by high content imaging analyses.

### Enhancing contractile structures and functions of iCMs by stoichiometric optimization of GHMT expression

**Figure 3:**
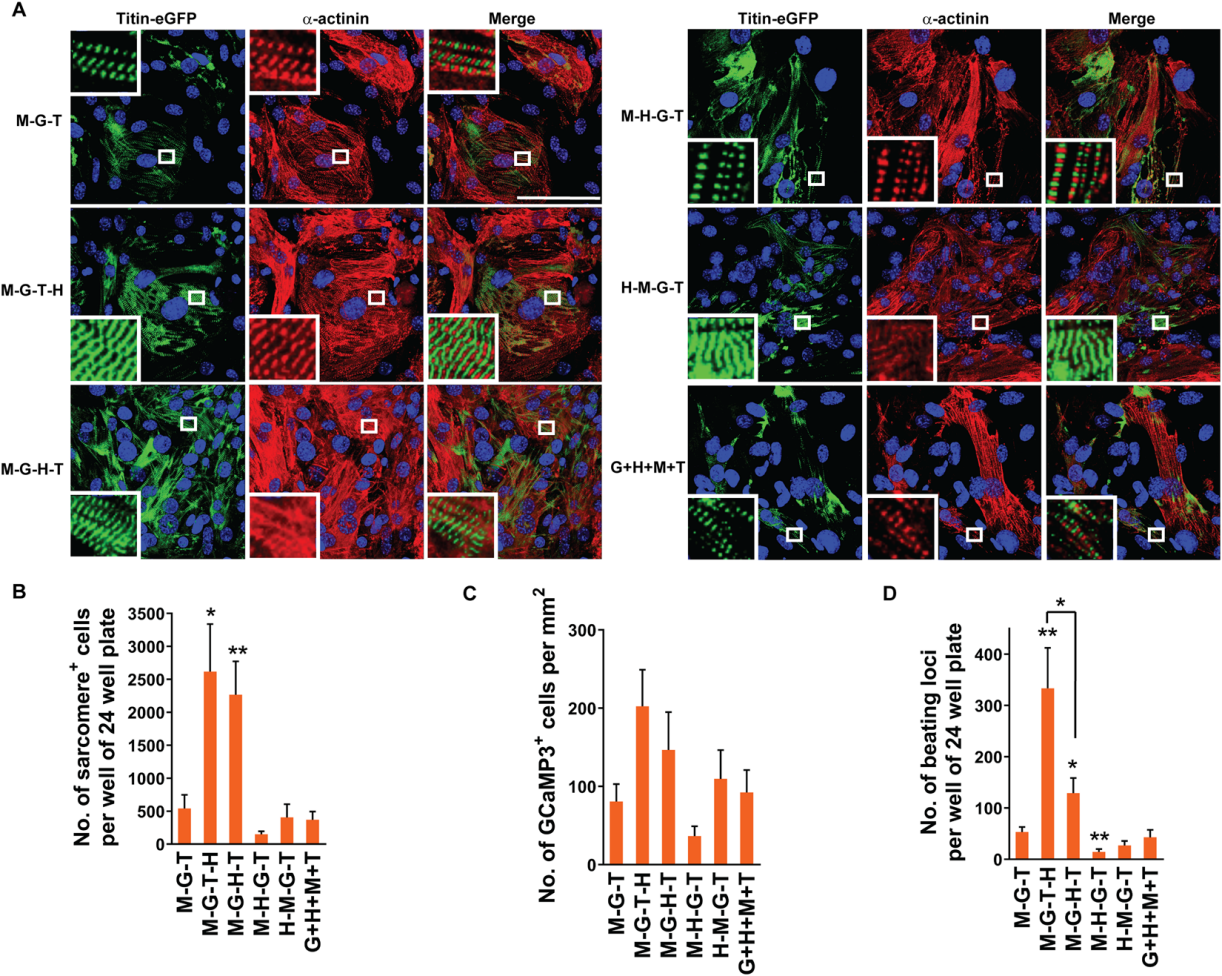
Structural and functional quality of the iCMs generated by different protein levels of cardiogenic transcription factors. (**A**) Visualization of sarcomere formation by immunofluorescence staining for Titin-eGFP and α-actinin following indicated transductions. The immunostained cells used for high content analyses were visualized using a 40X objective of a confocal microscope. White boxes are enlarged in insets to demonstrate M-band (Titin-eGFP) or Z-disk (α-actinin) structures in the sarcomere. Scale bar, 100 µM. (**B**) Quantification of well-organized sarcomere^+^ cells identified by visualizing M-band structures with Titin-eGFP expression following indicated transductions. Well-organized sarcomeric structures were counted using a 40X objective of an epifluorescence microscope. Eight independent experiments are presented as mean ± s.d. *P < 0.05; **P < 0.01 versus M-G-T. (**C**) Quantification of GCaMP3^+^ cells following indicated transductions into MEFs isolated form αMHC-Cre: Rosa26-GCaMP3 mice. Calcium oscillation identified by flashing green fluorescence was counted using a 20X objective of an epifluorescence microscope at day 18 post-transduction. Four independent experiments are presented as mean ± s.d. See also Movies S1–6. (**D**) Quantification of spontaneously beating loci following indicated transductions. Beating loci were counted at day 18 post-transduction. Six independent experiments are presented as mean ± s.d. *P < 0.05; **P < 0.01 versus M-G-T unless specifically indicated. See also Movies S7–12.

Given that building a basic contractile unit, a sarcomere, is essential for generating functionally active cardiomyocytes by reprogramming^4^, we further examined sarcomere assembly of directly reprogrammed cells by forced expression of different levels of GHMT or previously optimized expression of GMT using the M-G-T construct^8^. We found that M-G-T-H and M-G-H-T transduction markedly increased the number of fibroblasts which developed organized sarcomere structures over transduction of other quad-cistronic constructs or the M-G-T tri-cistronic construct (Fig. 3A,B). We were able to visualize well-organized sarcomeric structures by demonstrating two distinct, non-overlapping, sarcomeric structures (i.e. M-band by Titin-eGFP and Z-disk by α-actinin) (Fig. 3A, white inlets). The relative effect of M-G-T-H or M-G-H-T transduction on sarcomere organization was more prominent than sarcomeric protein induction, demonstrating ~4–5-fold increase over transduction of M-G-T or other constructs (Fig. 3B). These results not only re-emphasize indispensable roles of Hand2^5^, but also reveal the importance of controlled relative protein levels of all four factors (i.e. GHMT), for contractile structure development during fibroblast to cardiomyocyte reprogramming.

Next, we analyzed the ability of calcium handling of iCMs using genetically encoded green fluorescent protein-based calcium indicator GCaMP. Since cardiomyocyte contraction is tightly coupled with intracellular calcium dynamics, spontaneous calcium flux induction is a necessary component for establishing a contractile phenotype of iCMs^13^. We transduced MEFs from the mice carrying αMHC-Cre and GCaMP3 alleles (αMHC-Cre: Rosa26-GCaMP3 mice) with four different quad-cistronic constructs, the M-G-T construct, or a mixture of individual constructs encoding each factor. In αMHC-Cre: Rosa26-GCaMP3 mice, expression of a calcium indicator, GCaMP3, is driven by activation of cardiac specific αMHC promoter^14,15^. We visualized oscillation of real-time GCaMP3 fluorescence and quantified GCaMP3 positive cells (Fig. 3C and Movies S1–6). We found that M-G-T-H transduction was more effective to induce spontaneous calcium oscillation by GCaMP3 than other polycistronic constructs or simultaneous GHMT transduction.

Spontaneous contraction is a unique functional property of a cardiomyocyte, requiring both organized contractile structures and calcium handling machineries. As a final proof of functionality of iCMs, we quantified spontaneous beating cells following transduction of four different quad-cistronic constructs, the tri-cistronic M-G-T, or four individual constructs encoding each factor together. M-G-T-H transduced cells showed most spontaneous beating cells, increasing the number of beating cells by ~6-fold and ~2.5-fold over M-G-T and M-G-H-T transduced cells, respectively (Fig. 3D and Movies S7–12). The significant difference in the number of beating cells between M-G-T-H and M-G-H-T transduction was noteworthy, since the differences in other cardiac phenotypes between two groups were modest without reaching a statistical significance (i.e. sarcomeric protein induction efficiency, the number of sarcomere^+^ cells and the number of GCaMP^+^ cells). Moreover, M-G-T-H transduction started to induce beating iCMs around D11 post-transduction, earlier than transductions of other constructs did (e.g. M-G-T and M-G-H-T: ~D12-13; M-H-G-T, H-M-G-T, and G + H + M + T: ~D15). In addition, we directly compared the ability of M-G-T-H transduction for inducing beating iCMs with that of M-G-T + H transduction. We found that M-G-T-H transduction induced about three fold higher number of spontaneous beating iCMs than M-G-T + H transduction (Fig. S3 and Movies S13–18), although the ability of M-G-T-H for sarcomere protein induction in fibroblasts was only modestly better than M-G-T + H transduction (Fig. S2). These results highlight that developing contractile cardiac phenotypes in fibroblasts requires more precise control of protein expression levels of all four reprogramming factors than inducing sarcomere proteins in fibroblasts. To demonstrate a direct mechanistic relationship between sarcomeric organization and contractility of iCMs, we visualized real-time spontaneous contractions of M-band labeled by Titin-eGFP following reprogramming of *Titin-eGFP* reporter MEFs (Movies S19–22). Collectively, our findings indicate that combinatorial expression of relatively lower level of Hand2 with higher level of Mef2c facilitates the reprogramming progress from induction of sarcomeric proteins to development of contractile structures and functions.

### Differential effects of stoichiometric alteration of GHMT expression on gene expression profile

**Figure 4:**
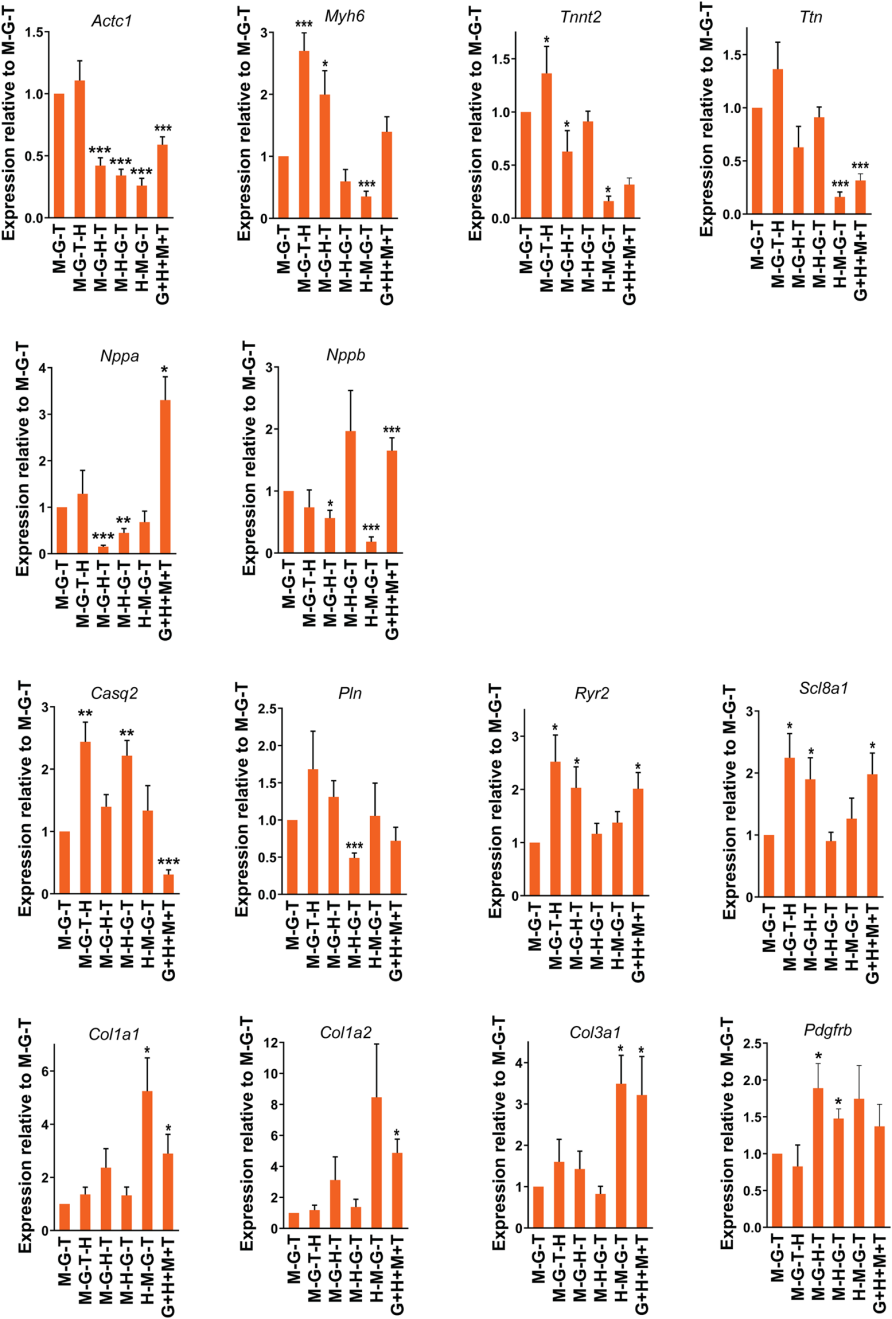
Gene expression profile in fibroblasts expressing different protein levels of cardiogenic transcription factors. Expression of cardiac and fibroblast genes induced by indicated transductions was quantified by qPCR 3 weeks post-transduction and normalized to the level induced by M-G-T transduction. Six or eight independent experiments are presented as mean ± s.d. *P < 0.05; **P < 0.001; ***P < 0.0001 versus M-G-T.

We examined how different expression levels of GHMT affect gene expression profile in directly reprogrammed cells. We performed qPCR analysis using a set of cardiomyocyte or fibroblast genes. We analyzed gene expression profiles in MEFs transduced with M-G-T tri-cistronic vector, four different quad-cistronic vectors, or individual vectors of GHMT by normalizing them to uninfected MEFs (Fig. S4). We also directly compared gene expression profiles by M-G-T transduction with those by other transductions leading to different expression levels of GHMT (Fig. 4). Regardless of relative GHMT or GMT protein expression levels, forced expression of GHMT or GMT by transduction of polycistronic or monocistronic constructs significantly increased expression of cardiac genes and decreased expression of fibroblast genes, as shown in previous studies^1,8,16^ (Fig. S4). Only M-G-T-H transduction showed higher or similar expression levels of all four sarcomere genes (i.e. *Actc1, Myh6*, *Tnnt2, and Ttn*), compared to M-G-T transduction (Fig. 4). Gene expression levels of cardiac peptides, *Nppa* and *Nppb*, were not correlated with the extent of cardiomyocyte reprogramming determined by structures and functions of iCMs in Figs 2 and 3, consistent with the previous study^8^. Although biological significance of this finding is unclear, it may suggest that very high expression of a couple of reprogramming factors may be sufficient to activate *Nppa* or *Nppb* expression. Overall, M-G-T-H transduction was most effective to upregulate expression of the genes associated with calcium handling in cardiomyocytes (i.e. *Casq2*, *Pln, Ryr2, and Slc8a1*). On the other hand, expression of fibroblast genes (i.e. *Col1a1, Col1a2, Col1a3*, and *Pdgfrb*) was markedly down-regulated regardless of relative protein levels of reprogramming factors, compared to uninfected MEFs (Fig. S4). Overall, H-M-G-T or uncontrolled transduction seemed to be less effective for silencing fibroblast genes, while M-G-T or M-G-T-H transduction was more consistent to repress all fibroblast genes tested than other transductions (Figs S4 and 4). Taken together, our data demonstrated that stoichiometric optimization of GHMT protein expression can augment activation of a new cardiac gene program, and at the same time repression of existing fibroblast gene program in fibroblasts.

## Discussion

The previous study showed that a precise control of GMT protein expression is more effective for re-directing a fibroblast fate toward a cardiac fate than uncontrolled expression of highest possible protein level of each factor^8^. Recently, we directly compared the reprogramming ability of GHMT with GMT using a selected fibroblast population where expression of GHMT or GMT proteins was confirmed^5^. While GHMT showed similar sarcomere protein induction efficiency to GMT expression, GHMT expression dramatically increased the number of fibroblasts adopting contractile structures and functions over GMT. Combining the results of these two studies, we reasoned that a precise control of GHMT proteins would further enhance the cardiomyocyte reprogramming process. Therefore, we sought to determine optimal stoichiometry of GHMT proteins for cardiomyocyte reprogramming using 2A peptide-based polycistronic vectors in this study. We found that M-G-T-H transduction inducing relatively lower Hand2 and higher Mef2c protein levels along with tightly controlled Gata4 and Tbx5 levels enhances cardiomyocyte reprogramming efficiency and quality over transductions of other quad-cistronic vectors or previously defined M-G-T tri-cistronic vector. Importantly, optimization of stoichiometric expression of GHMT markedly enhanced organization of myofibrillar structures, calcium handling, and contractility in iCMs, while it modestly increased sarcomere protein induction efficiency, compared to stoichiometrically optimized GMT or uncontrolled GHMT expression. These results suggest that the stoichiometric effect of GHMT expression culminates in unhindered progresses from sarcomeric protein induction toward development of contractile structures and functions in fibroblasts. Our results highlight that developing functionally active contractile structures requires more precise control of each reprogramming factor’s protein expression than simply inducing cardiac proteins in fibroblasts. Furthermore, we demonstrated that M-G-T-H transduction significantly enhanced reprogramming efficiency and quality over simply combining M-G-T with Hand2 transduction. These results point to the importance of stoichiometric expression of Hand2 along with that of GMT for cardiomyocyte reprogramming, and provide further evidence that ensuring expression of all four reprogramming factors in individual cells enhances cardiomyocyte reprogramming^5^. However, it is important to note that a significant population of M-G-T-H transduced fibroblasts still fail to develop contractile structures and functions, suggesting that optimizing stoichiometric protein expression of GHMT alone is unlikely to be sufficient for fibroblast to functional iCM reprogramming. It will be worth identifying additional genetic or epigenetic requirements that govern the induction process for full-blown cardiac phenotypes within the context of optimized stoichiometric GHMT expression in fibroblasts. In addition, it remains to be determined whether cardiomyocyte reprogramming of human fibroblasts or *in vivo* reprogramming in a mouse disease model could be improved by optimized stoichiometric expression of GHMT. This study not only increases our understanding of minimal genetic requirements for fibroblast to contractile iCM conversion, but also provides a new standard platform for further optimizing cardiomyocyte reprogramming. We expect that the effects of previously identified genetic or pharmacological reprogramming boosters could be maximized when the stoichiometric requirement for GHMT expression is achieved in fibroblasts.

## Methods

### Animals

All animal procedures performed in this study were approved by the Institutional Animal Care and Use Committee at Vanderbilt University Medical Center. All experimental procedures were performed in accordance with the relevant guidelines and regulations. *Titin-eGFP* reporter knock-in mice were generous gifts from Dr. Gotthardt^9^.

### Plasmids

Monocistronic retroviral vectors encoding mouse *Gata4, Hand2, Mef2c*, and *Tbx5* were previously generated^16^. To generate four quad-cistronic constructs encoding GHMT, pGEMT-easy vectors harboring M-G-T and tdTomato provided by Dr. Liu (i.e. M-*P2A*-G-*T2A*-Tbx5-*E2A*-tdTomato, M-*P2A*-G-*T2A*-tdTomato-*E2A*-*Tbx5*, M-*P2A*-tdTomato-*T2A*-G-*E2A*-Tbx5, and tdTomato-*P2A*-M-*T2A*-G-*E2A*-Tbx5)^7^ were used as templates. tdTomato was excised and replaced with myc-tagged *Hand2* in pGEMT vector. Then, the four quad-cistronic expression cassettes within pGEMT vectors were excised and individually subcloned into pBabe-X retroviral vectors.

### Isolation of mouse embryonic fibroblasts

MEFs were isolated as described previously^5^. The head, extremities, internal organs of the chest and abdominal cavities were carefully removed from E13.5 or E14.5 mouse embryos. After being minced, the remaining mouse embryonic tissues were incubated with 0.25% trypsin for 15 min at 37 °C water bath. After filtering the digested tissues through a cell strainer, the collected cells were expanded with fibroblast growth medium containing 10% FBS and 1% penicillin/streptomycin for 24–48 hours. The harvested MEFs were counted and frozen at −80 °C overnight, and stored at a liquid nitrogen tank until they were used.

### Generation of retroviruses

Retroviruses were generated as described previously^5^. pBabe-X retroviral constructs were transfected into Platinum E cells (Cell Biolabs) at 30–40% confluency using Fugene 6 (Promega). At 16–20 hours post-transfection, transfected Platinum E cells were replenished with the fresh growth medium (DMEM with 10% FBS and 1% penicillin/streptomycin). At 48 hours post-transfection, the viral medium was filtered through a 0.45 µm polyethersulfone (PES) filter. Pre-counted frozen MEFs (2–3 × 10^4^ cells/a well) were plated into a 24 well black clear bottom plate (Greiner, cat# 662892) 16–20 hours prior to transduction. The viral medium mixed with Polybrene at a concentration of 6 µg/ml replaced the fibroblast growth medium in the cell culture plate. The same transfection was independently repeated for generating the second viral medium 24 hours after the first transfection. The second viral medium replaced the first viral medium in the culture plate with MEFs. Twenty-four after the second infection, MEFs were replenished with the cardiac induction medium containing A-83-01 (Tocris, cat# 2939) and SB431542 (Sigma, cat# S4317) as described previously^5^. The induction medium was changed every 3 days until cells were analyzed.

### Immunocytochemistry

Immunocytochemistry was performed as described previously^5^. Cells were fixed with 2% paraformaldehyde for 15 min followed by permeabilization with permeabilization buffer (0.05% Triton-X in PBS) for 5 min three times at room temperature. Following incubating with blocking buffer (Universal blocking buffer, BiogeneX, cat# HK083-50K) for 45 min at room temperature, cells were incubated with primary antibodies against α-actinin (Mouse monoclonal, Sigma, cat# A7811, 1:400 dilution) and GFP (Chicken IgY fraction, Invitrogen, cat# A10262, 1:400 dilution) overnight at 4 °C. Following washing with permeabilization buffer three times for 5 min, cells were incubated with Alexa fluorogenic secondary antibodies (Invitrogen) at 1:400 dilution at room temperature for 1 hour. Cells were washed again three times with permeabilization buffer for 5 min, and then incubated with Hoechst solution at a final concentration of 2 µM in permeabilization buffer for 15 min. After cells were washed three more times, cell images were captured with a Zeiss LSM 500 confocal microscope or ImageXpress Micro XL Automated Cell Imaging system (Molecular Device).

### High content imaging and analysis

High content imaging analysis was performed as described previously^5^. The same immunostained cells used for immunocytochemistry imaging were re-used for high content imaging analysis. Each 24 well plate contained a control well with uninfected cells which were immunostained same as other wells. Immunostained cells were analyzed with ImageXpress Micro XL Automated Cell Imaging system (Molecular Device). Cell images were acquired with a 10X objective at 25 or 36 fields per well. DAPI, Texas Red, and FITC filter sets were used to detect Hoechst, α-actinin, and Titin-eGFP, respectively. While exposure time for nuclear Hoechst staining was automatically set by autoexposure, exposure times for Texas Red and FITC filter sets were manually optimized to the level which allows the minimum number of false positive cells in the control well of each 24 well plate. Twenty-five or thirty-six captured images per each 24 well were analyzed with MetaXpress software (Molecular Device) to quantify the number of Texas Red and/or FITC fluorescent positive cells among DAPI positive cells.

### Western blotting

Western blotting was performed as described previously^5^. The cell lysates were collected using RIPA buffer (Sigma) and run on 4–20% gradient or 10% SDS-PAGE gel (Bio-Rad). After proteins were transferred to PVDF membrane (Bio-Rad), immunoblotting was performed using primary antibodies against GATA4 (Rabbit polyclonal, Thermo Scientific, cat# PA1-102), Myc (Rabbit polyclonal, Santa-Cruz Biotechnology, cat# sc-789, for detecting myc-tagged HAND2), Mef2c (Rabbit polyclonal, GeneTex, cat# GTX 105433), Tbx5 (Rabbit polyclonal, Proteintech, cat# 13178-1-AP), and GAPDH (Mouse monoclonal, Santa Cruz Biotechnology, cat# sc-365062). Protein expression levels were quantified using Image J.

### Flow cytometry

Flow cytometry analysis was performed as described previously with minor modifications^16,17^. Cells were collected with 0.125% trypsin and fixed with fixation buffer (BD Bisoscience, cat# 554655) for 20 min on ice. Fixed cells were washed with Perm/Wash buffer (BD Bisoscience, cat# 554723) once. Then, cells were incubated with mouse monoclonal anti-cTnT antibody (Thermo Scientific, cat# MA5-12960) at 1:200 dilution in Perm/Wash buffer for 1.5 hr at room temperature. Following washing with Perm/Wash buffer, cells were incubated with goat anti-mouse Alexa fluor 647 (Invitrogen) at 1:400 for 1 hr. After another Perm/Wash buffer wash, cells were re-suspended with stain buffer (BD Bisoscience, cat# 554656), and then analyzed for cTnT expression using FACS Caliber (BD sciences) and FlowJo software.

### Quantification of spontaneously beating iCMs

Transduced cells in a well of a 24 well plate were examined under an inverted microscope (Leica DMIL LED Inverted Microscope with fixed stage) using 10X objective. Beating loci were manually counted throughout the whole field of each well. Counting of beating loci was repeated three times for each well. The average number was presented as a number of beating loci for an individual experiment.

### Quantitative real time PCR (qPCR)

Three weeks after transduction of indicated retroviral vectors, total RNA was extracted from the transduced MEFs and uninfected MEFs as a control using NucleoSpin RNA Kit (Macherey-Nagel). cDNA was synthesized by reverse transcription qPCR using High-Capacity cDNA Reverse Transcription Kit (Applied Biosystems). qPCR experiments were performed with SYBR probes and iTaq Universal SYBR Green Supermix (Bio-Rad) using a Bio-Rad CFX96 system (Bio-Rad).

### Statistical analyses

Statistical significance was determined using unpaired two-tailed Student’s t-test. P-values of < 0.05 were regarded as significant.

## Supplementary information

Supplementary Figures and Figure Legends

Movie S1

Movie S2

Movie S3

Movie S4

Movie S5

Movie S6

Movie S7

Movie S8

Movie S9

Movie S10

Movie S11

Movie S12

Movie S13

Movie S14

Movie S15

Movie S16

Movie S17

Movie S18

Movie S19

Movie S20

Movie S21

Movie S22

## Data Availability

The datasets generated during and/or analysed during the current study are available from the corresponding author on reasonable request.
