# Supplementary material for: Stoichiometric optimization of Gata4, Hand2, Mef2c, and Tbx5 expression for contractile cardiomyocyte reprogramming
Source: Sci Rep. 2019 Oct 18;9:14970. doi: 10.1038/s41598-019-51536-8 (PMC6800441; doi:10.1038/s41598-019-51536-8)
Supplement: Supplementary file 1 — Supplementary Figures and Figure Legends [file 41598_2019_51536_MOESM1_ESM.docx]

**Stoichiometric optimization of Gata4, Hand2, Mef2c, and Tbx5 expression for contractile cardiomyocyte reprogramming**

Zhentao Zhang^1, 2, 3^, Wenhui Zhang^1, 2, 3^, and Young-Jae Nam^1, 2, 3, *^

^1^Department of Medicine, Division of Cardiovascular Medicine, Vanderbilt University Medical Center, Nashville, TN, USA

^2^Department of Cell and Developmental Biology, Vanderbilt University, Nashville, TN, USA

^3^Vanderbilt Center for Stem Cell Biology, Vanderbilt University, Nashville, TN, USA

*To whom correspondence may be addressed:

Young-Jae Nam, M.D., Ph.D.

Department of Medicine, Division of Cardiovascular Medicine

Vanderbilt University Medical Center

Nashville , Tennessee 37232, USA

Phone: 615-936-5436

E-mail: [young-jae.nam@vanderbilt.edu](mailto:young-jae.nam@vanderbilt.edu)

**Supplementary Figures and Figure Legends**

**
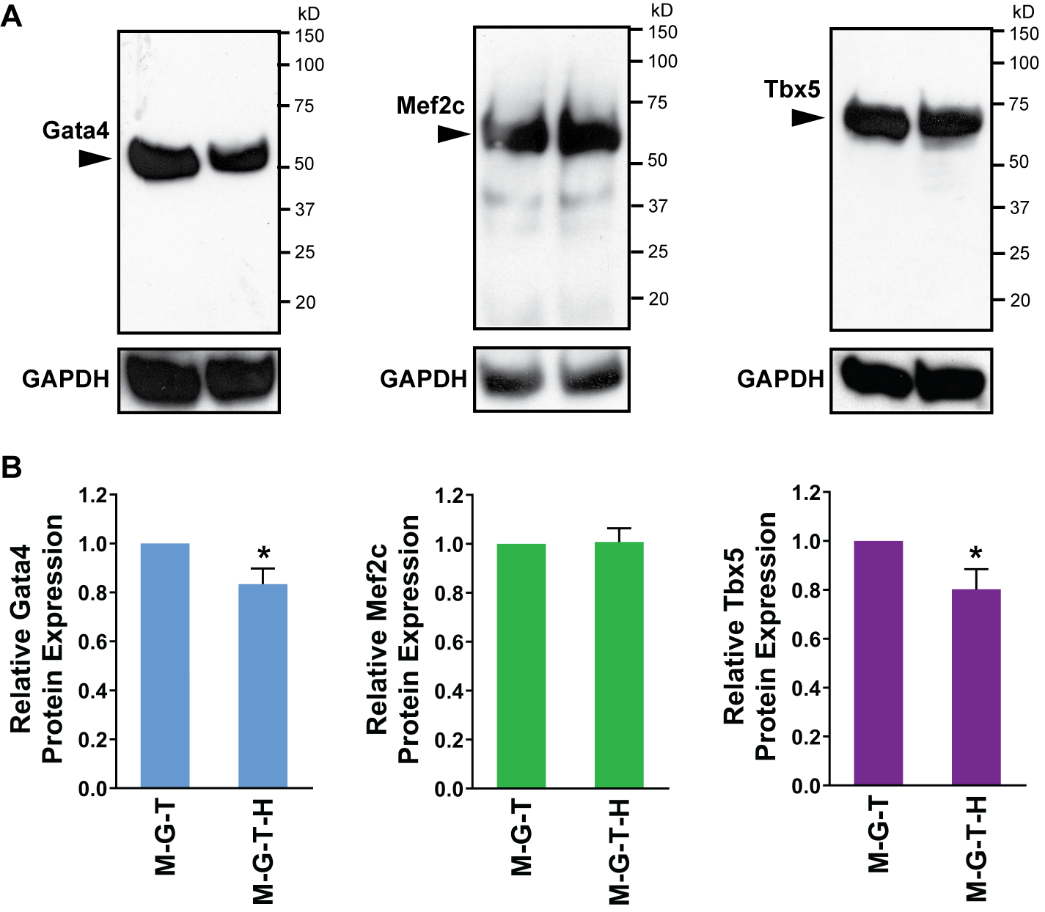
**

**Figure S1**. Comparison of Gata4, Mef2c, and Tbx5 protein expression levels between M-G-T and M-G-T-H transductions. (A) Western blot analysis for GMT protein expression. Three days after transducing M-G-T or M-G-T-H vector into MEFs, cell lysates were collected. An arrow head indicates a protein band of each cardiogenic transcription factor. (B) Quantification of relative GMT protein expression levels. Seven independent experiments are presented as mean+s.d. **P*<0.05.


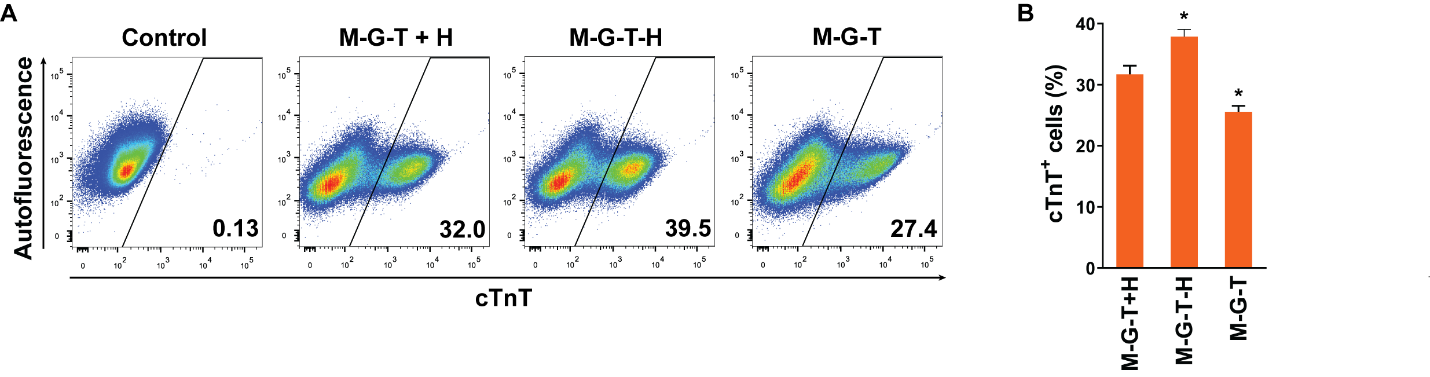


**Figure S2**. Comparison of sarcomere protein induction efficiency by M-G-T + H with M-G-T-H or M-G-T. (A) Representative flow cytometry plot for analyzing cTnT expressing cells. Wild type MEFs were transduced with indicated vectors. At D15 post-transduction, the percentage of cTnT^+^ cells were analyzed by flow cytometry. (B) Summary of flow cytometry analyses. Data from three independent experiments are presented as mean+s.d. **P*<0.05 versus M-G-T + H.

**
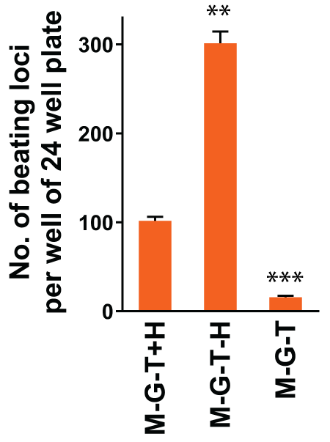
**

**Figure S3**. Comparison of the spontaneously beating loci number between M-G-T + H and M-G-T-H or M-G-T transduction. Beating loci were counted at day 18 post-transduction. Three independent experiments are presented as mean+s.d. ***P*<0.001, ****P*<0.0001 versus M-G-T + H. See also Supplementary Movie 13-18.

**
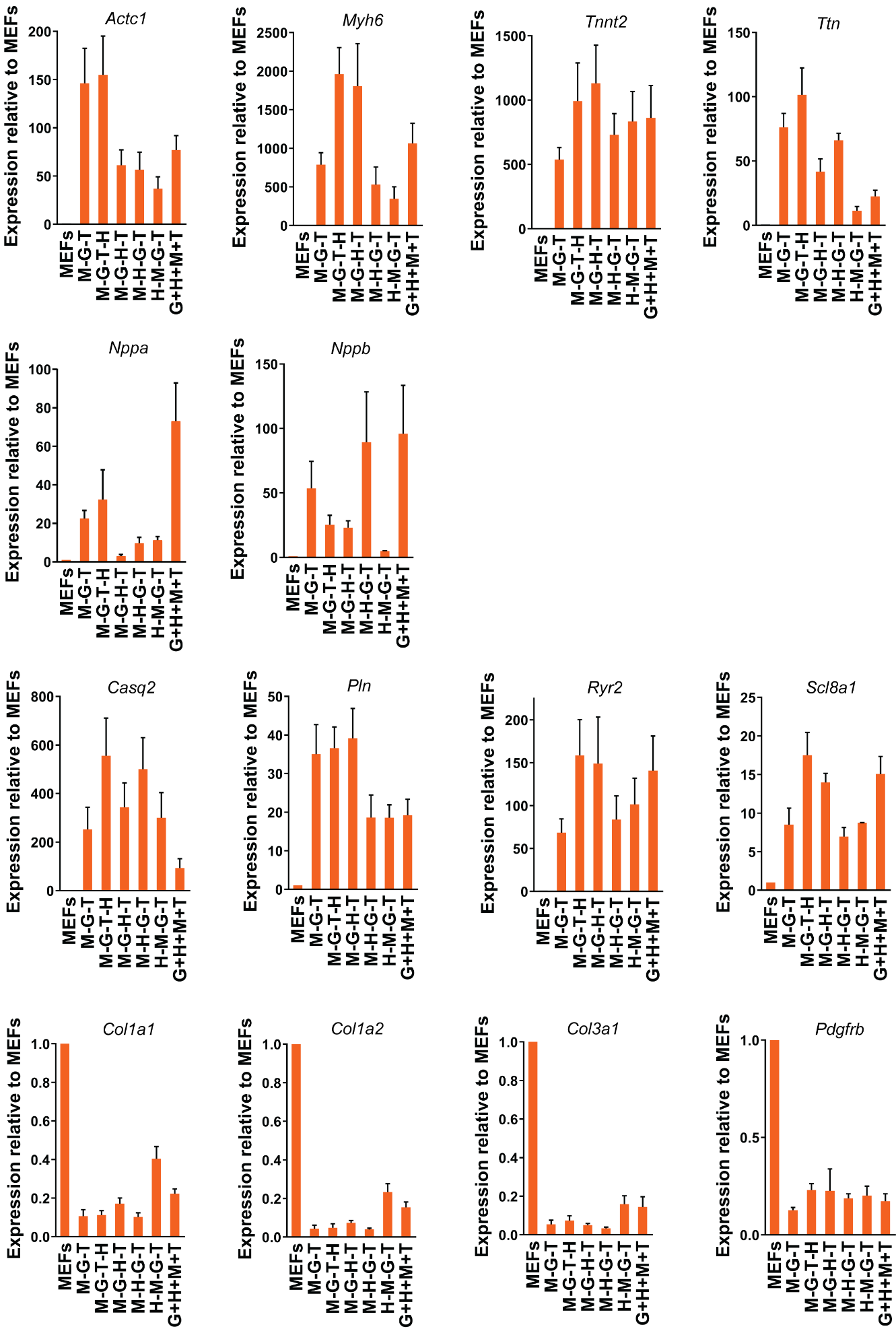
**

**Figure S4**. Gene expression analyses in fibroblasts transduced with M-G-T tri-cistronic vector, four different quad-cistronic vectors, or individual vectors of GHMT. Expression of cardiac and fibroblast genes was quantified by qPCR 3 weeks post-transduction and normalized to uninfected MEFs. Six or eight independent experiments are presented as mean+s.d.

**TITLES AND LEGENDS FOR SUPPLEMENTARY MOVIES**

**Movie S1-6**. Calcium oscillation visualized by GCaMP3 reporter. Related to Figure 3C. MEFs isolated from αMHC-Cre: Rosa26-GCaMP3 mice were transduced with the indicated constructs (Movie S1: M-G-T, Movie S2: M-G-T-H, Movie S3: M-G-H-T, Movie S4: M-H-G-T, Movie S5: H-M-G-T, and Movie S6: G+H+M+T). At day 18 post-transduction, GCaMP expressing cells were visualized using an epifluorescence microscope.

**Movie S7-12**. Spontaneously beating iCMs induced by M-G-T-H, M-G-H-T or M-G-T transduction. Related to Figure 3D. MEFs isolated from wild type mice were transduced with the M-G-T-H (Movie S7-S8), M-G-H-T (Movie S9-S10), or M-G-T (Movie S11-S12) polycistronic construct. At day 18 post-transduction, spontaneously beating cells were visualized using a 20X microscope objective.

**Movie S13-18**. Spontaneously beating iCMs induced by M-G-T+H, M-G-T-H or M-G-T transduction. Related to Supplementary Fig. 3. MEFs isolated from wild type mice were transduced with the M-G-T + H (Movie S13-S14), M-G-T-H (Movie S15-S16), or M-G-T (Movie S17-S18) construct. At day 18 post-transduction, spontaneously beating cells were visualized using a 10X (S13, S15, and S17) or 20X (S14, S16, and S18) microscope objective.

**Movie S19-22**. Spontaneous contraction of M-band sarcomeric structures. MEFs isolated from *Titin-eGFP* reporter knock-in mice were transduced with the M-G-T-H (Movie S19 and S20) or M-G-H-T (Movie S21 and S22) quad-cistronic construct. At day 18 post-transduction, spontaneously beating cells were identified. Then, spontaneous contractions of M-band structures labeled by Titin-eGFP were visualized using a 40X objective of an epifluorescence microscope.
